# Mast cell degranulation and bradykinin-induced angioedema - searching for the missing link

**DOI:** 10.3389/fimmu.2024.1399459

**Published:** 2024-05-15

**Authors:** Grzegorz Porebski, Alicja Dziadowiec, Hubert Rybka, Radoslaw Kitel, Mateusz Kwitniewski

**Affiliations:** ^1^ Department of Clinical and Environmental Allergology, Jagiellonian University Medical College, Krakow, Poland; ^2^ Department of Immunology, Faculty of Biochemistry, Biophysics and Biotechnology, Jagiellonian University, Krakow, Poland; ^3^ Doctoral School of Exact and Natural Sciences, Jagiellonian University, Krakow, Poland; ^4^ Department of Organic Chemistry, Faculty of Chemistry, Jagiellonian University, Krakow, Poland

**Keywords:** bradykinin, C1 inhibitor deficiency, HAE, hereditary angioedema, mast cell, MRGPRX2

## Abstract

Initiation of the bradykinin generation cascade is responsible for the occurrence of attacks in some types of angioedema without wheals. Hereditary angioedema due to C1 inhibitor deficiency (HAE-C1-INH) is one such clinical entity. In this paper, we explore the existing evidence that mast cells (MCs) degranulation may contribute to the activation of the kallikrein-kinin system cascade, followed by bradykinin formation and angioedema. We present the multidirectional effects of MC-derived heparin and other polyanions on the major components of the kinin-kallikrein system, particularly on the factor XII activation. Although, bradykinin- and histamine-mediated symptoms are distinct clinical phenomena, they share some common features, such as some similar triggers and a predilection to occur at sites where mast cells reside, namely the skin and mucous membranes. In addition, recent observations indicate a high incidence of hypersensitivity reactions associated with MC degranulation in the HAE-C1-INH patient population. However, not all of these can be explained by IgE-dependent mechanisms. Mast cell-related G protein-coupled receptor-X2 (MRGPRX2), which has recently attracted scientific interest, may be involved in the activation of MCs through a different pathway. Therefore, we reviewed MRGPRX2 ligands that HAE-C1-INH patients may be exposed to in their daily lives and that may affect MCs degranulation. We also discussed the known inter- and intra-individual variability in the course of HAE-C1-INH in relation to factors responsible for possible variability in the strength of the response to MRGPRX2 receptor stimulation. The above issues raise several questions for future research. It is not known to what extent a prophylactic or therapeutic intervention targeting the pathways of one mechanism (mast cell degranulation) may affect the other (bradykinin production), or whether the number of mast cells at a specific body site and their reactivity to triggers such as pressure, allergens or MRGPRX2 agonists may influence the occurrence of HAE-C1-INH attacks at that site.

## 1 Introduction

Despite years of research into angioedema, particularly hereditary angioedema due to C1-inhibitor deficiency, some clinical aspects of the disease remain unclear, such as the wide clinical variation in disease severity between members of the same family carrying the same causative mutation, or the precise mechanism that triggers the angioedema at a given site (1, 2). These issues are still under investigation. In the past, authors studying the pathomechanisms of HAE-C1-INH have repeatedly pointed to possible links between the bradykinin-generating cascade observed in HAE-C1-INH and mast cell degranulation.

**Figure 1:**
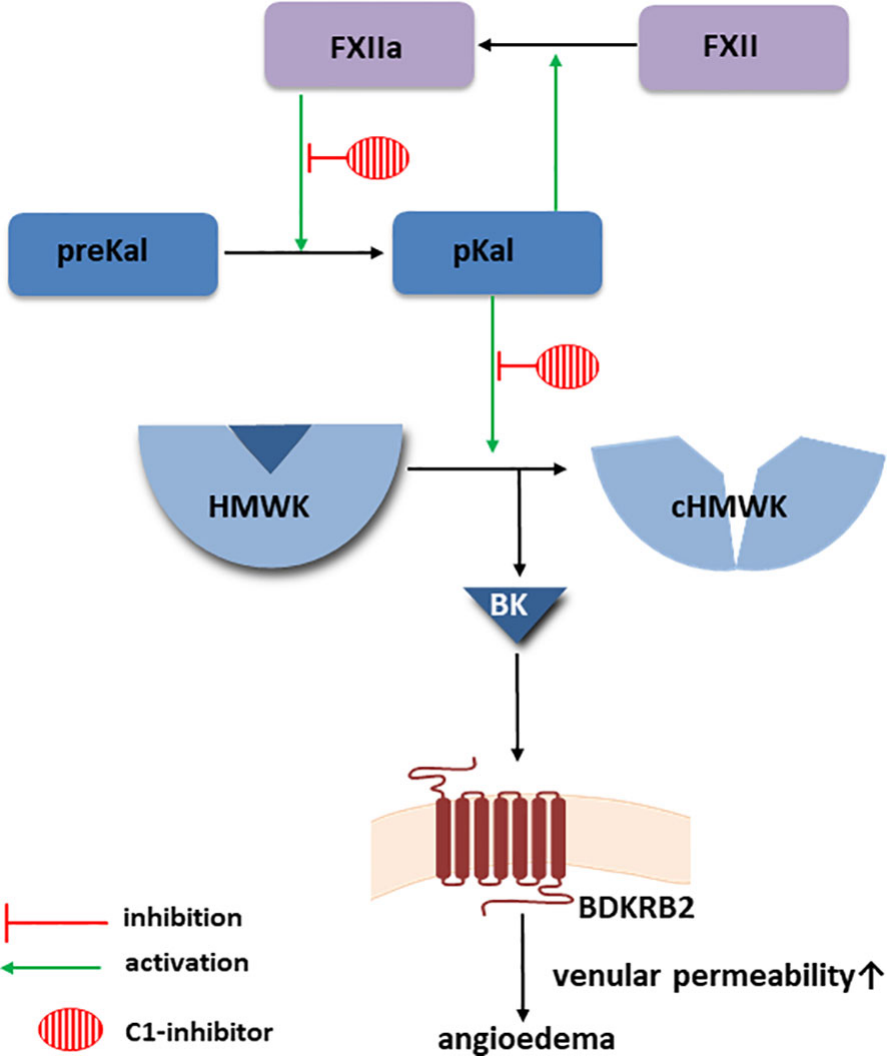
Bradykinin-forming cascade. Created with Motifolio (Motifolio Inc., Elliocott City, MD, USA) BDKRB2, Bradykinin receptor B2; BK, bradykinin; cHMWK, cleaved HMWK; FXII, factor XII; FXIIa, activated FXII; HMWK, high molecular weight kininogen; MC, mast cells; pKal, plasma kallikrein; preKal, prekallikrein.

Kaplan recently suggested that in cases of anaphylaxis accompanied by hypotension and laryngeal edema, the activation of the bradykinin-forming cascade, which is also responsible for the development of angioedema in HAE-C1-INH, may be the important contributing factor (3, 4). The same author also emphasizes the potential significance of mast cell (MC)-derived heparin in the activation of the factor XII (FXII), which is also present during HAE-C1-INH attacks (5). Other authors have noted the connection between bradykinin production and mast cell activation during allergic reactions. They cite data from animal models and clinical observations of patients with allergic reactions induced by food and insect venom (6). However, they emphasize that the evidence of such a relationship is sparse and that further research is necessary. The authors of another review paper on the links between mast cells and the contact system are of a similar opinion (7). They describe the potential involvement of mast cell heparin, proteoglycans, polyphosphates, and other active substances produced in MCs in initiating the activation of the kallikrein-kinin system cascade, starting with factor XII (**Figure 1**). The authors emphasise that under *in vivo* conditions, mast cells are the exclusive site of heparin synthesis. Above findings correspond with recently published observations of Farkas et al. (8), who showed that hypersensitivity reactions induced primarily by common allergens occur approximately three times more frequently in the HAE-C1-INH patient population than in the general population.

Recent years have brought new insights into the structure and function of mast cell. The work by McNeil et al. has generated great interest in the Mas-related G protein-coupled receptor-X2 (MRGPRX2) (9). Using a knockout mouse model and cell lines, they demonstrated that MRGPRX2 represents an alternative pathway to the classical IgE-dependent MC activation pathway and can be stimulated not only by its natural endogenous ligands, but also by a large group of exogenous compounds. This has drawn attention to IgE-independent mechanisms of mast cell activation, particularly in drug hypersensitivity reactions (10) and chronic spontaneous urticaria, where MRGPRX2 was found to be upregulated in skin mast cells of patients with severe course of disease (11). This in turn led to clinical trials of an oral synthetic MRGPRX2 antagonist (12). Although data on the clinical significance of MRGPRX2 are still relatively sparse, it has already been mentioned in a new classification of hypersensitivity reaction types (13). Depending on the activation pathway, mast cells respond by secreting different sets of mediators (14), which may result in a distinct effector response to their stimulation. In the case of MRGPRX2-dependent activation, degranulation is rapid and occurs through small single granules as demonstrated by Gaudenzio et al. (15).

In this article, we explore the potential links between mast cell degranulation and bradykinin-induced angioedema, both as observed in the clinic and in laboratory and molecular studies.

## 2 Interactions between MC degranulation and bradykinin formation

Research on *in vitro* contact system activation processes dates back many years. Such studies have shown that heparin glycosaminoglycans and proteoglycans from some natural sources, such as rat mast cells and porcine mucosa, activate the conversion of prekallikrein to plasma kallikrein, and that the reaction is determined by the negative charge of these macromolecules (16). Subsequent studies using mast cell heparin proteoglycan (MC-HepPG) and other glycosaminoglycans have shown that, like dextran sulphate, they cause activation of the FXII ultimately leading to the cleavage of high molecular weight kininogen (HMWK) (17). Moreover, the addition of heparinase I and II to the experimental system inhibited such a MC-HepPG-dependent reaction. However, some other substances tested (from the group of sulfated polysaccharides) did not exhibit similar activity. The authors suggest that their results may explain the occurrence of activation of the contact system after exposure to an allergen. This phenomenon has been observed in a subsequent study of patients allergic to insect venom, who underwent anaphylaxis after stinging (18). In most of these patients, the increases in plasma levels of C1-inhibitor with the factor XIIa (FXIIa) and with kallikrein complexes, and levels of cleaved HMWK were observed after in-hospital venom challenge. The increases were particularly pronounced in patients with angioedema, whereas they did not occur in healthy controls and in patients who did not develop symptoms after venom challenge.

The findings from the above reports were later supported by an analysis of adverse reactions that occurred in patients treated with heparin in hemodialysis facilities. It should be noted that heparin used as an anticoagulant differs from heparin derived directly from mast cells. The former has little or no effect on FXII-dependent bradykinin formation, whereas mast cell heparin, due to a relative excess of sulphation, has properties that can activate the bradykinin-forming cascade. It turned out that patients who received heparin contaminated with oversulfated chondroitin sulfate (OSCS) developed typical symptoms consistent with hypersensitivity, including a drop in blood pressure, nausea, shortness of breath, but also swellings, most often of the lips, eyelids, and throat (19). An explanation for this phenomenon has been provided by a study which demonstrated that OSCS found in contaminated heparin, as well as OSCS obtained synthetically, showed the ability to activate the kinin-kallikrein system *in vitro*, leading to bradykinin production (20). Moreover, this effect was also confirmed by *in vivo* experiments in pigs, which responded with kallikrein activation and hypotension after intravenous administration of OSCS. Shortly after, another group showed that reduced levels of C1-inhibitor are associated with an increased risk of the above-mentioned reactions after OSCS (21). On the other hand, some authors have reported that negatively charged heparin can modify the interaction of serine proteases with their inhibitors, such as kallikrein and C1-inhibitor (22). They suggest that heparin may enhance kallikrein inhibition by potentiating C1-inhibitor activity, which could be used for therapeutic purposes in HAE-C1-INH. However, attempts to use this as a therapeutic intervention have yielded inconclusive results (23, 24).

A further important contribution to this topic was provided by the study by Oschatz et al. (25). The authors provided extensive evidence in a number of experimental settings that mast cell-derived heparin increases vascular permeability by generating bradykinin secondary to activation of factor XII. They used both *in vitro* experiments with human plasma and animal models, and finally clinical observations in HAE-C1-INH patients. Another study in a mouse model found that deficiency or inhibition of the kinin-kallikrein system (at different levels from factor XII to the bradykinin B2 receptor) attenuated the mast cell response to allergen exposure, suggesting that bradykinin is also involved in the effector response initiated by the IgE-dependent pathway (26). Subsequently, activation of the contact system was also observed in the plasma of patients during acute anaphylaxis symptoms, confirming the findings from the animal model in this study. Similar observations were made in another study in patients with chronic spontaneous urticaria (CSU) (27), in which mast cell degranulation also occurs, as it happens in anaphylaxis. Cleaved HMWK levels were significantly elevated in symptomatic patients with CSU compared to healthy controls, and were similar to levels observed in patients with HAE-C1-INH. These results indicate that bradykinin production is increased in urticaria, however the magnitude is much less then with anaphylaxis and of questionable clinical relevance.

## 3 Clinical links between mast cells-dependent angioedema and hereditary angioedema

In principle, bradykinin-mediated angioedema (hereditary angioedema due to C1 inhibitor deficiency) and angioedema induced by histamine from degranulated mast cells are distinct clinical phenomena. However, they share some common features, such as occurring in the skin and mucous membranes (28), so in areas where mast cells are localized (14, 29). They may also be triggered by similar factors (**Table 1**). Many factors that are considered clinically relevant mast cell activators, such as bacterial components, physical stimuli, cold, pressure (38) are also known triggers of HAE-C1-INH attacks. However, it should be noted that these factors may not be equally important in both situations. Additionally, when comparing these triggers, it is important to consider the subjective nature of the information provided by patients. They may tend to focus on factors they believe are associated with inducing symptoms, which may result in overestimation of the importance of certain factors and the overlooking of others (28). Common features of different types of angioedema were sought by Schulkes et al. (39). They studied a group of patients with angioedema, including those with wheals, idiopathic angioedema, and ACE-I-induced angioedema, focusing on clinical characteristics, location, and potential triggers of symptoms. The authors summarized their findings by stating that the similarities in the clinical picture observed between the analyzed groups of angioedema suggest the presence of common pathomechanisms. However, it should be noted that angioedema in patients with CSU never includes laryngeal edema, which can be present when bradykinin is involved, for example in HAE-C1-INH (40).

**Table 1:** Common triggers of HAE attacks and corresponding triggers of different types of urticarial.

Hereditary angioedema	Urticaria
physical exertion, prolonged sitting or standing (30, 31)	physical exercise (cholinergic urticaria) (32)
psychological stress (30, 33–36)	stress (32)
mechanical trauma, nontraumatic tissue compression, exposure to cold (30, 31, 33–37)	physical factors (delayed pressure urticarial, cold urticarial, vibratory angioedema, others) (32)
infections (30, 31)	infections (32, 35)
foodstuffs, food (30, 33, 36, 37)	food allergy in sensitized patients, nonallergen food components (32, 35)
insect bites, Hymenoptera stings (30, 36)	insect stings, Hymenoptera stings (38)
drugs: ACE-I (30, 33), others* (36)	drugs: NSAIDs (32), others (NMBAs, FQ, opioids)* (35)

*rare or poorly documented; ACE-I, angiotensin-converting enzyme inhibitors; NSAIDs, non-steroidal anti-inflammatory drugs; NMBA, neuromuscular-blocking agents; FQ, fluoroquinolones.

It is estimated that HAE-C1-INH attack triggers are reported by about 1/3 to 90% of patients (35). Therefore, direct triggers for the remaining HAE-C1-INH attacks are unknown. Reported triggers for HAE-C1-INH attacks include such factors as foods, foodstuff and insect venoms (**Table 1**). Typically, effects of these triggers are associated with IgE-mediated mechanisms inducing hypersensitivity reactions. In the case of HAE-C1-INH, IgE dependence often cannot be confirmed, as described by Steiner et al. in their cohort of patients (36). Grumach et al. note that the mechanism underlying activation of the kinin-kallikrein system is not clear in cases of such triggers, and the absence of an IgE-specific response in HAE-C1-INH patients reporting these triggers suggests that another, non-IgE-dependent mechanism induces symptoms (35). One possible explanation for these observations is the presence of substances in food or insect venoms that induce mast cells degranulation via an IgE-independent pathway, such as MRGPRX2. Activated mast cells would then be expected to release heparin and other mediators, thereby contributing to the activation of the kinin-kallikrein system and, consequently, to the induction of angioedema. However, not all patients exposed to potential triggers develop symptoms. In the following section, we address the question of whether HAE-C1-INH patients are exposed to MRGPRX2 ligands, if they are related to triggers of HAE-C1-INH attacks, and what may account for the variability in response to potential stimulation with them.

## 4 MRGPRX2 triggering and its individual variability as an example of a factor influencing mast cells degranulation

MRGPRX2 has been shown to be activated by a wide range of exogenous ligands including insect venoms and host defence peptides, molecules and toxins released by bacteria, a wide range of plant-derived organic compounds and commonly used substances (41). Currently, the ever-increasing number of plant constituents (comprising mostly ingredients of herbal medicines and dietary substances) have been widely identified as MRGPRX2 agonists or antagonists (**Table 2**). However, evidence for interactions between MRGPRX2 and these ligands is mostly based on *in vitro* studies in cell lines, *in vivo* mouse models of anaphylaxis, and in silico molecular docking (41, 77).

**Table 2:** Examples of exogenous MRGPRX2 ligands.

Ligand	Chemical structure	Source	Activity towards MRGPRX2	References
INSECT-DERIVED COMPOUNDS				
Mastoparan	peptide	wasp venom	agonist	(42, 43)
P17	peptide	ant venom	agonist	(44)
Mast cell degranulating peptide	peptide	bee venom	agonist	(45)
*Automeris zaruma* venom	not identified	caterpillar venom	agonist	(46)
IP defensin 1	peptide	tick salivary	agonist	(47)
BACTERIA-DERIVED COMPOUNDS				
Staphylococcus δ-toxin	peptide	*Staphylococcus aureus*	agonist	(48)
Competence stimulating peptide-1	peptide	*Streptococcus pneumoniae*	agonist	(49)
FOODSTUFFS and DIETARY SUPPLEMENTS				
Quercetin	flavonoid	fruits and vegetables	antagonist	(50)
Genistein	flavonoid	legumes	antagonist	(51)
Liquiritin	flavonoid	licorice (sweets)	antagonist	(52)
Licorice chalcone A	chalconoid	licorice (sweets)	antagonist	(53)
Isoliquiritigenin	chalconoid	licorice (sweets)	antagonist	(54)
Fisetin	flavonoid	fruits and vegetables	antagonist	(55)
Kaempferol	flavonoid	fruits and vegetables	antagonist	(56)
Caffeic acid phenethyl ester	polyphenol	coffee, wine	antagonist	(57)
Curcumin	polyphenol	*Curcuma longa*	antagonist	(58)
Rosmarinic acid	polyphenol	*Perilla frutescens, Rosmarinus officinalis*	antagonist	(57, 59, 60)
Piperine	alkaloid	*Piper longum*, *Piper nigrum*	antagonist	(61)
HERBAL MEDICINES				
Baicain	flavonoid	*Scutellaria baicalensis*	agonist	(62)
Apigenin	flavonoid	*Perilla frutescens*	antagonist	(59)
Imperatorin	coumarin	*Angelicae Dahuricae*	antagonist	(63)
Osthole	coumarin	*Cnidium monnieri*	antagonist	(64)
Hydroxysafflor yellow A	alkaloid	*Carthamus tinctorius*	antagonist	(65)
Isosalvianolic acid C	polyphenol	*Salvia miltiorrhi*	agonist	(66, 67)
Peoniflorin	glycoside	*Paeoniae alba*	antagonist	(68)
Saikosaponin A	glycoside (saponin)	*Perilla frutescens*	antagonist	(59, 69)
Ginsenosides	glycoside (saponin)	*Schisandra chinensis*	agonist	(70)
Celastrol	quercetin galactoside	*Tripterygium wilfordii*	antagonist	(71)
Mucunain	cysteine protease	*Mucuna pruriens*	agonist	(72)
OTHER				
Sinomenine	alkaloid	*Caulis sinomenii*	agonist	(73–75)
Thebaine	alkaloid	*Pupuver* *somniferurn*	agonist	(73)
Thiomersal	preservative	e.g. vaccines, cosmetics, tattoo inks	agonist	(76)

There are many reasons for the individual variability in the expression or structure of MRGPRX2. Genetic factors have been widely discussed, primarily focusing on single nucleotide polymorphisms (SNPs) located within the protein coding region of the *MRGPRX2* gene (41, 78). Dozens of SNPs located within the coding region of the human *MRGPRX2* locus are known (79). Notably, both loss-of-function (75, 80, 81) and gain-of-function SNPs have been identified in the MRGPRX2 gene, with the latter resulting in enhanced degranulation upon stimulation with the receptor ligand (82). Importantly, small insertion/deletion polymorphisms and SNPs located outside of the protein coding region have not yet been investigated. SNPs located within the promoter region of a gene may influence the binding of transcription factors and affect promoter activity, DNA methylation, and histone modifications (83–90). SNPs in the introns can affect RNA splicing and can promote or disrupt the binding and function of long non-coding RNAs (lncRNAs) (91–93). SNPs located in the 5’- and 3’- untranslated regions (UTRs) may affect protein translation and miRNA-dependent gene silencing (94, 95). These aforementioned mechanisms may potentially impact the expression level of MRGPRX2 and lead to the production of variants with different properties. However, to date, only two transcript variants of MRGPRX2 have been described (96), both of which encode the same protein. Epigenetic processes, including DNA methylation, histone modification, and various RNA-mediated processes, represent another potential source of variability in MRGPRX2 expression (97). Methylation status, even at a single CpG locus, can modulate protein expression (98). However, this area remains largely unexplored in terms of its influence on MRGPRX2.

Alternatively, MRGPRX2 expression may vary between individuals or within a single individual due to changes in the local tissue microenvironment. Mast cells, the primary cells expressing MRGPRX2, located in different layers or zones within a tissue, may encounter unique microenvironmental cues, such as variations in oxygen tension, nutrient availability, or interactions with neighbouring cells, which may impact their phenotype. The variability of MRGPRX2 mRNA expression in the skin samples from healthy individuals is pronounced, with a high coefficient of variation (102.9%) (99). Our preliminary studies have shown that MRGPRX2 expression in mast cells is dynamically regulated by factors released from both healthy and psoriatic skin tissues (100). It has also been demonstrated that patients with chronic urticaria have a significantly higher number of MRGPRX2 positive skin mast cells and a higher percentage of MRGPRX2 positive mast cells compared to control subjects (101). In addition, codeine acts through MRGPRX2 (102) and response to codeine is accentuated in patients with CSU (103).

Interestingly, icatibant, which is used in the on-demand treatment of acute HAE-C1-INH attacks due to its blockade of the bradykinin B2 (BDKRB2) receptor, has been also demonstrated to trigger MRGPRX2 (9, 104). Both receptors belong to the family of G protein-coupled receptors (GPCRs) and share common structural features with other class A GPCRs, such as canonical seven-transmembrane (TM) helices, a conserved disulfide bond between TM3, and extracellular loop 2 (ECL2) and helix 8 lying parallel to the plasma membrane (105–109). The extracellular regions and their closest transmembrane regions are responsible for ligand binding, while the intracellular regions and their closest TM regions are involved in G protein coupling and downstream signalling (106, 109). Ligands for BDKRB2 include bradykinin and Lys-bradykinin (110), as MRGPRX2 can be activated by numerous ligands with cationic properties, which are endogenous and exogenous compounds with diverse chemical features (77). However, it remains unclear to what extent the other MRGPRX2 ligands, in addition to icatibant, can trigger or inhibit BDKRB2.

## 5 Mast cell heparin and other polyanions as a modulators of the major components of the kinin-kallikrein system

Mast cell heparin and other polyanions act as a modulators of the major components of the contact system. The modification of the activity of the components of the contact system occurs through a charge neutralisation mechanism (111). Mast cell heparin is able to bind a wide variety of proteins (112). However, the effects of mast cell heparin binding vary significantly between proteins that form contact system. In this way, mast cell heparin and other polyanions can provide a balance between activation and inhibition of certain pathways within the contact system (111, 113). For instance, when mast cell heparin potentiates the activity of C1-inhibitor, which acts as a major negative regulator of FXIIa and plasma kallikrein, it leads to the inhibition of the kallikrein-kinin system and reduction of bradykinin release (111). Conversely, activation of factor XII by mast cell heparin and other polyanions (113) initiates a cascade of bradykinin release, as discussed below.

FXII is secreted into the bloodstream in its inactive (zymogen) form and constitutes a single-chain glycoprotein of 596 amino acid residues (∼80 kDa). The zymogen is converted to its active form, termed α-FXIIa, by proteolytic cleavage of the R353-V354 peptide bond, resulting in the formation of two separate protein chains that remain connected by a disulfide bond. The cleavage occurs either in a process of autoactivation or, more efficiently, upon interaction with plasma kallikrein (114). However, it is known, that in order for this conversion to occur, FXII must first be bound to a negatively charged surface or molecule. A variety of agents have been identified that allow FXII activation, ranging from exogenous substances such as glass and kaolin clay to organic molecules such as dextran sulphate (115), oversulfated chondroitin sulfate (OSCS) (20), extracellular RNA (116), or endogenous polyphosphate ions released by activated platelets (117). Although the critical role of these negatively-charged polyanionic molecules or surfaces is clear, the exact mechanism by which FXII activation occurs is still under debate.

FXII is a multi-domain protein and does not appear to have an exclusive site for polyanion binding. Apart from its catalytic domain (called the light chain upon conversion to FXIIa), which is formed by the C-terminal sequence, six major domains can be identified towards the N-terminus; a proline-rich region, a kringle domain, two fibronectin and two EGF-like domains (118). Upon activation of FXII, these domains form what is known as the heavy chain. A strong argument for the crucial role of the N-terminal domains in polyanion binding was provided by Citarella and colleagues (119), who determined that recombinant FXII lacking either three or five of the heavy chain domains had a significantly lower affinity towards glass and dextran sulphate.

**Figure 2:**
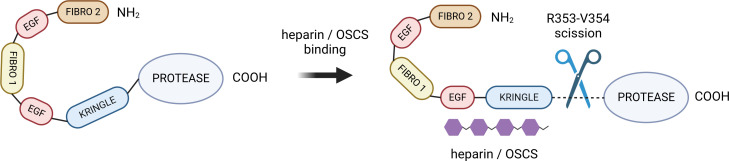
The hypothesized mechanism of heparin/OSCS-mediated FXII activation. The negatively charged polysaccharide binds to kringle and EGF-like domains, inducing a conformational charge that exposes the R353-V354 cleavage site (116). Created with BioRender.com. OSCS, oversulphated chondroitin sulphate.

A unique insight into the possible mechanism of FXII activation was offered by de Maat (120), who hypothesised that several distinct binding sites for different subtypes of anionic surfaces exist among the above-mentioned domains. This claim was supported by the observation that the binding of polyanions does not always result in FXII activation, and a specific binding mode, and thus, a specific protein conformation is needed for FXII to perform its biological function. In particular, binding to EGF-like and kringle domains has been described as a strong requirement for the activation of FXII, which is a binding mode exhibited by dextran sulphate according to Citarella (119). This led de Maat to formulate a conformation-dependent FXII activity model, according to which the conversion to the active α-FXIIa form is only attainable upon binding of some N-terminal domains to a polyanionic surface or polymer, forcing a conformational change that exposes the crucial R353-V354 cleavage site (see **Figure 2**). It is important to note, however, that while the binding of most sulfate-rich polysaccharides seems to make FXII susceptible to cleavage by plasma kallikrein, the long-chain saccharides are capable of inducing autoactivation upon binding. This claim is based on a study by Silverberg et al. (121) who showed that FXII undergo autoactivation in the presence of dextran sulphate of molar ratio 500,000, whereas fractions with lower molar ratio values give very low rates of autoactivation. Heparin, having typically between 3,000 and 30,000 Da (122), can promote cleavage by plasma kallikrein, which falls in line with the experimental data (123). As suggested by the conformation-dependent activity model, full extension of the FXII heavy chain, facilitated by long anionic polysaccharides or solid surfaces, may be crucial for the autoactivation to occur.

The interactions between FXII and sulfate-rich polysaccharides appear to be primarily electrostatic in nature. The heavy, polyanion-binding chain (residues 1-353) contains an unbalanced excess of lysine and arginine residues, and is therefore positively charged, with a theoretical isoelectric point of 8.82 as determined by the Expasy ProtParam tool (124). It is tempting to hypothesize that polysaccharides with greater negative charge density will form tighter bonds with the heavy chain, and that oversulfated chondroitin sulfate will therefore activate factor XII more efficiently than heparin. There is also evidence that OSCS have an affinity for FXIIa (125). Activated FXII can then cleave its substrates and initiate a cascade of bradykinin release. As OSCS also bind to HMWK (125), it is conceivable that the affinity of OSCS for both α-FXIIa and HMWK may increase the rate of this reaction by directing the two proteins together.

## 6 Summary

Above, we discussed the abundant evidence demonstrating a link between mast cell degranulation and activation of the bradykinin generation cascade. We presented the multidirectional effects of mast cell heparin and other polyanions on the major components of the kinin-kallikrein system and pointed out some clinical similarities between HAE-C1-INH and mast cell degranulation in urticaria, with regard to the triggers of symptoms. We also recalled a publication that found that patients with HAE-C1-INH are more likely to report hypersensitivity reactions and have more swelling attacks during the pollen season (8). These are not the only such observations, as similar ones were made by Swedish authors studying the prevalence of allergy, asthma and atopic dermatitis in the HAE-C1-INH patient population (126). However, further in-depth studies are needed to verify these observations, as planned by Horváth et al. in a continuation of their work (8). Assuming the existence of a link between mast cell degranulation and HAE-C1-INH symptoms, one might expect that manifestations of hypersensitivity reactions favor angioedema attacks, and therefore HAE-C1-INH is more easily diagnosed in these patients than in C1-inhibitor deficient patients without hypersensitivity reactions. In this context, we addressed the issue of inter- and intra-individual causes of variability in the course of HAE-C1-INH.

Further, we used MRGPRX2 as an example of a factor that has a modulating effect on mast cell activation. It has recently attracted considerable interest in the scientific community (9, 41, 77, 78, 99, 105), as it has been recognized as a underlying mechanism for many mast cell degranulation-related reactions, the mechanism of which was unclear. Although, as we mentioned above, most of the data on MRGPRX2 comes from *in vitro* studies, there are also ongoing clinical trials using agonist of this receptor in chronic spontaneous urticaria and atopic dermatitis (12). There are many ligands in the everyday environment to which HAE-C1-INH patients may be exposed that can both activate and inhibit MRGPRX2 (**Table 2**), which in turn may lower or raise the threshold for triggering mast cell degranulation, respectively. In addition, there are many potential factors responsible for possible variability in the strength of the response to MRGPRX2 receptor stimulation, ranging from microenvironmental conditions to SNPs, as discussed above. Mast cells themselves also exhibit a high degree of heterogeneity in terms of the distribution and sets of mediators secreted under given conditions (14, 29), adding to the variability in potential clinical responses.

This raises several questions for future research. We do not know the extent to which prophylactic or therapeutic intervention targeting the pathways of one mechanism (mast cell degranulation) may affect the other (bradykinin production) (3). Will drugs that block the action of bradykinin help control anaphylactic reactions (3), or will drugs that block mast cell degranulation, such as omalizumab (11) or MRGPRX2 antagonists (12), reduce HAE-C1-INH attacks? Is the localization of mast cells in subcutaneous tissue and mucous membranes related to the localization of HAE-C1-INH attacks? To what extent does the number of mast cells at a particular site and their responsiveness to triggers such as pressure, allergens or MRGPRX2 agonists affect the occurrence of HAE-C1-INH attacks at that site? Although a seminal report by Nusseberg et al. showed that plasma bradykinin levels were elevated in HAE-C1-INH and angiotensin-converting enzyme inhibitor-induced edema, bradykinin was also detected at lower levels in the plasma of patients with histaminergic angioedema (127). It cannot be excluded that clinically relevant activation of the contact system only occurs in very severe reactions associated with mast cell degranulation, such as anaphylaxis (4, 18), but bradykinin is already generated with less massive MC degranulation. Such degranulation may contribute to the induction of angioedema attacks in individuals with reduced C1-inhibitor activity (21). The relationship between the clinical course and the pathomechanisms of HAE-C1-INH requires further research. The hypotheses discussed in this article suggest some areas for further research, which are listed in **Table 3**.

**Table 3:** Implications of the potential link between mast cell degranulation and activation of the bradykinin generation cascade for future research questions.

• Clinical picture
✔ Can mast cell degranulation triggers influence the initiation of bradykinin-dependent angioedema, in particular HAE-C1-INH? ✔ Does bradykinin play a role in anaphylaxis with associated upper airway angioedema? ✔ Does individual mast cells localisation in the patient’s body correlate with the localisation of HAE-C1-INH attacks? ✔ Are mast cell-dependent diseases relevant to the course of HAE-C1-INH?
• MRGPRX2 as an example of a potential cause of variability in the course of HAE-C1-INH outside the kinin-kallikrein system
✔ To what extent does the genetic background of a given factor matter (e.g. SNPs, epigenetic processes)? ✔ To what extent does the expression of a given factor matter (e.g. individual variability, changes in the local tissue microenvironment)?
• Therapeutic interventions
✔ Can inhibition of mast cell degranulation affect HAE-C1-INH? ✔ Can bradykinin inhibition improve the outcome of anaphylaxis with laryngeal oedema?

## Data availability statement

The original contributions presented in the study are included in the article/supplementary material. Further inquiries can be directed to the corresponding author.

## Author contributions

GP: Conceptualization, Funding acquisition, Supervision, Validation, Writing – original draft, Writing – review & editing. AD: Writing – original draft, Writing – review & editing. HR: Writing – original draft, Writing – review & editing. RK: Writing – original draft, Writing – review & editing. MK: Writing – original draft, Writing – review & editing.
